# Editorial on polygenic risk scores: Colloquium held at the Centre for Personalised Medicine, Oxford

**DOI:** 10.1017/pcm.2023.22

**Published:** 2023-10-05

**Authors:** Padraig Dixon, Sarah Briggs, Anneke Lucassen

**Affiliations:** 1 Nuffield Department of Primary Care Health Sciences, University of Oxford, Oxford, UK; 2 Centre for Personalised Medicine, St Anne’s College, University of Oxford, Oxford, UK; 3 Nuffield Department of Medicine, University of Oxford, Oxford, UK; 4 Clinical Ethics, Law and Society, Wellcome Centre for Human Genetics, University of Oxford, Oxford, UK

**Keywords:** Risk factors, personalized medicine, polygenic risk

Polygenic risk scores (PRSs) have generated substantial interest in personalised or precision medicine circles in recent years, with enthusiasm from industry and public sectors due to their potential to provide insights into an individual’s disease susceptibility. Unlike rare diseases where a few genetic variants often dominate a risk profile, PRS amalgamate the effects of numerous genetic variants. These variants individually may have minor impacts on disease risk, but collectively they enable an assessment of genetic susceptibility to common multifactorial diseases such as heart disease and cancer.

By adding PRS to conventional risk assessment tools (e.g. the QRISK algorithm to assess cardiovascular disease risk used in UK general practice), it is argued that better predictions can be made to inform population health strategies. These might include screening programmes, preventative interventions, and lifestyle advice, and the hope is that this would lead to improved population-wide outcomes. The effectiveness of (in particular) screening programmes depends on how well the risk assessment discriminates between individuals who will develop the disease and who will not, as well as the ability of subsequent interventions to influence the course of the disease.

Those who argue that PRS will improve existing screening approaches often appear to sit at odds with those who emphasise the need to address several key challenges before considering their widespread integration into healthcare. The Centre for Personalised Medicine (CPM), University of Oxford, in conjunction with *Cambridge Prisms: Precision Medicine* facilitated a discussion at this intersection to locate the common ground in this debate. We convened a colloquium entitled “Opportunities & challenges for polygenic risk scores in healthcare: Can we find common ground?”

The colloquium consisted of an introduction, and round-up, from CPM team members, three talks focussing on particular disease areas, and a panel discussion with audience participation. Dr. Amit Sud (Institute of Cancer Research) discussed the opportunities of using PRS in common forms of cancer (breast, colon and prostate in particular). Prof. Aroon Hingorani (University College London) outlined issues in cardiovascular disease and Prof. Cathryn Lewis (Kings College London) discussed potential neuropsychiatric applications.

This was followed by a panel discussion, chaired by Prof. Clare Turnbull (Institute of Cancer Research), involving the audience and contributions from the three case study speakers together with Prof. Nick Wald (University College London), Dr. Judith Hayward (NHS England – North East and Yorkshire), Prof. Claudia Langenberg (Queen Mary University of London) and Dr. Imran Rafi (St George’s, University of London). The talks, and excerpts of the debate, are recorded on the CPM website (http://cpm.ox.ac.uk; Centre for Personalised Medicine, 2023).

One key area of common ground was that the clinical value of a particular PRS is dependent on the risks and benefits of the intervention that follows as a result of such testing. If the subsequent intervention is definitive and low risk, a PRS that allows its better targeting would be welcomed. Similarly, if PRSs can be used to reduce the frequency of screening, or allow less invasive screening to those at lower risk of a disease, then this is another potential benefit. Where there is no evidence-based intervention to offer after screening – some neuropsychiatric conditions for example- then predicting low or higher PRSs for a condition may, at best, support nosology but may have limited alternative uses at present.

There was also consensus that the prediction accuracy of PRSs is limited because common genetic variation comprises only a small part of the overall risk of common conditions like heart disease or cancer. Most of the susceptibility to these diseases is explained by environmental or stochastic processes, so even if all genetic factors that carry any risk can be identified and incorporated in a PRS, their predictive ability will be limited. For example, Zhang et al. (2020) showed that a breast cancer PRS with a specificity (the ability of the PRS test to correctly identify true negatives) of 95%, the highest attainable sensitivity (the ability of PRS to correctly predict disease onset) would be 19%, some 4% greater than current methods. This means most true positive cases would still be missed with a highly predictive hypothetical PRS.

Prof. Hingorani agreed that PRSs could improve risk prediction marginally, depending on context. He presented an example which examined the impact of screening 100,000 people for cardiovascular disease using conventional risk factors, and conventional risk factors plus a PRS. With a 10% 10-year risk cut-off, the number needed to genotype to find an additional case was 1,149, while the number needed to genotype to prevent an additional CVD event was 5,882. By contrast, simply offering statins to all adults aged 40 years and older had a 10-year number needed to prevent a CVD event of just 63.

Prof. Lewis noted that there are potential uses of PRS in the prediction of the course of a disease, and in informing nosology. Polygenic risk for major depression increases suicide risk across psychiatric disorders (Mullins et al., 2019), and may also allow differentiation between schizophrenia-spectrum disorder, bipolar disorder and psychotic depression following a first episode of psychosis (Rodriguez et al., 2023). PRSs may also contribute to understanding pharmacogenetic response (Zhai et al., 2022).

The ensuing debate was wide-ranging, focused primarily on screening and risk stratification using PRSs, and covered some of the issues that need to be considered in the adoption of PRS in healthcare. For example,

- Unlike most other biomarkers, genetic variants are fixed at conception and can be assayed at any point in life, potentially enabling screening or stratification from any age. For example, Richardson et al. (2022) note that PRSs, while currently limited, may allow the stratification of young children for cardiometabolic traits. At the same time, there are significant (practical and ethical) implications of labelling young children as having risks for health conditions that may not manifest themselves, if ever, for decades.
- A screening programme is usually only commissioned if it can reliably detect diseases at a point where an intervention will change the course of the disease. This is in part why, for example, prostate screening is problematic- many tumours remain indolent and would not affect survival but their detection may cause considerable anxiety and uncertainty about treatment. In this and similar cases, it is important that research identifies predictors of disease progress as well as disease onset.
- An important distinction between the clinical validity and utility of PRSs was also debated. The former asks whether PRSs can effectively stratify patients according to the risk of future disease whilst the latter assesses whether the PRS can improve patient outcomes. A recent review found that clinical utility was often missing in examples of PRS that had clinical validity (Kumuthini et al., 2022). For example, prostate cancer PRSs are associated with disease risk in trans-ethnic populations (Huynh-Le et al., 2022), but it does not necessarily follow that PRS-informed screening for prostate cancer would have clinical utility and indeed may result in net harm in the presence of over-diagnosis.
- Evidence on PRS cost-effectiveness is an important consideration for any intervention including screening programmes (Dixon et al., 2022). The costs of PRSs include not only the sample collection and genotyping costs, but also the downstream healthcare costs of clinical advice and follow-up investigations.
- There will also be trade-offs between different outcomes produced by PRS-informed risk stratification. For example, Dr. Sud considered the impact of offering annual mammography to women aged 40–50 with high or moderate polygenic risk for breast cancer (Sud et al., 2023). In the UK, population mammography screening starts at 50, but starting a decade earlier based on PRSs would detect 1,700 more cancers in the UK population, but would also result in 5,722 false positive results, and would still result in more than 4,000 cancer cases remaining unidentified. These 4,000 cases might have been reassured by their low PRSs and not been as vigilant of early symptoms. The acceptability of these types of trade-off to individuals and society requires further exploration.
- The General Practitioners on the panel emphasised other practical implications of introducing PRSs in practice, including the communication skills required to communicate these findings, particularly where many people’s expectation of genetic influences on disease may be over- or under-stated.

Finally, the broader context in which we make healthcare decisions is rapidly changing. For one, any emphasis on polygenic risk should not be at the expense of other influences on health. The over-representation of European ancestral backgrounds in genetic research is increasingly understood (Popejoy and Fullerton, 2016) and was noted as a significant challenge in our discussions, particularly as the majority of existing PRSs are most predictive for individuals of European ancestry. The use of PRSs in population-level programmes could have the inadvertent effect of widening avoidable health inequalities, although this remains a topic of much ongoing research (Ruan et al., 2022).

With the effects of climate change increasingly visible, many health systems have committed to achieving net-zero healthcare (Karliner et al., 2023), and mass genetic testing is likely to have a significant carbon footprint. Whether the environmental cost is sufficiently offset by the clinical and other benefits obtained is an area for further evaluation (e.g. if waste associated with the manufacture, distribution and administration of systemic therapeutics is reduced or avoided [Weadick et al., 2023]) and as well as the direct and indirect non-financial costs of these tests.

Further opportunities to debate these complex issues were welcomed, and a follow-up event focusing on a specific disease area is planned.
