# Supplementary material for: Editorial on polygenic risk scores: Colloquium held at the Centre for Personalised Medicine, Oxford
Source: Camb Prism Precis Med. 2023 Oct 5;1:e32. doi: 10.1017/pcm.2023.22 (PMC10953748; doi:10.1017/pcm.2023.22)
Supplement: Peer review history

# Editor report: Recommendation: Editorial on polygenic risk scores: Colloquium held at the Centre for Personalised Medicine, Oxford — R0/PR2

**DOI:** 10.1017/pcm.2023.22.pr2

**Affiliations:** Editorial Hub, United Kingdom of Great Britain and Northern Ireland

No accompanying comment.

# Editor report: Decision: Editorial on polygenic risk scores: Colloquium held at the Centre for Personalised Medicine, Oxford — R0/PR3

**DOI:** 10.1017/pcm.2023.22.pr3

**Affiliations:** Institute of Cardiovascular & Medical Sciences, University of Glasgow College of Medical Veterinary and Life Sciences, United Kingdom of Great Britain and Northern Ireland

No accompanying comment.
